# PREVALENCE OF HEARING SYMPTOMS RELATED TO PATULOUS EUSTACHIAN TUBE AFTER BARIATRIC SURGERY

**DOI:** 10.1590/0102-672020200002e1520

**Published:** 2020-11-20

**Authors:** Leticia Raysa Schiavon KINASZ, Hendrick Emmanuel Vieira DE-SOUSA, Manoel Augusto Ribas CAVALCANTI, José Fernando POLANSKI

**Affiliations:** 1 Otorhinolaryngology; 2 General Surgery, Hospital de Clínicas Complex, Federal University of Paraná, Curitiba, PR, Brazil

**Keywords:** Bariatric surgery, Eustachian tube, Otolaryngology, Cirurgia bariátrica, Tuba auditiva, Otolaringologia

## Abstract

**Background:**

Rapid and severe weight loss can result in the reduction of the ear tube lining fat tissue and it becomes patent, leading to symptoms such as autophony, aural fullness and tinnitus. Patients after bariatric surgery have, in theory, a predisposition to the development of such alteration.

**Aim:**

To evaluate the presence of patent tuba-related complaints in patients undergoing bariatric surgery, correlating with weight and body mass index (BMI) values, as well as demographic data.

**Methods:**

Cross-sectional study composed of the evaluation of patients undergoing bariatric surgery through a standardized questionnaire about the presence of symptoms compatible with ear tube patency.

**Results:**

Eighty patients were evaluated, 77 female and three males. The main comorbidity was systemic arterial hypertension (37.5%). Fifteen (18.75%) presented symptoms compatible with patent auditory/Eustachian tube - aural fullness and autophony - postoperatively. In symptomatic individuals the initial weight was 112 kg on average and the preoperative BMI was 45 kg/m², while in asymptomatic individuals the weight was 117 kg and BMI 47 kg/m². There was statistical significance in the comparison between individuals with and without symptoms in the variables of initial weight (p=0.00000), current weight (p=0.00029), preoperative BMI (p=0.00219) and postoperative BMI (p=0.00148).

**Conclusion:**

The presence of symptoms compatible with patent auditory/Eustachian tube was 18.75% of the patients submitted to bariatric surgery in the evaluated sample. Both preoperative weight and BMI were lower in symptomatic patients when compared with the asymptomatic group.

## RESUMO

**Racional:**

A perda ponderal rápida e intensa pode resultar na redução do tecido gorduroso de revestimento da tuba auditiva e ela se tornar patente, gerando sintomas como autofonia, plenitude aural e zumbido. Pacientes após operação bariátrica possuem, em teoria, predisposição para o desenvolvimento de tal alteração.

**Objetivo:**

Avaliar a presença de queixas relacionadas à tuba patente em pacientes submetidos à cirurgia bariátrica, correlacionando com os valores de peso, índice de massa corpórea e de dados demográficos.

**Método:**

Estudo transversal composto pela avaliação de pacientes submetidos à cirurgia bariátrica através de questionário padronizado, sobre a presença de sintomas compatíveis com patência de tuba auditiva.

**Resultados:**

Foram avaliados 80 pacientes, 77 mulheres e três homens. A principal comorbidade foi hipertensão arterial sistêmica (37,5%). Quinze (18,75%) apresentaram sintomas compatíveis com tuba auditiva patente - plenitude aural e autofonia - no pós-operatório. Nos sintomáticos o peso inicial era em média 112 kg e o IMC pré-operatório de 45 kg/m²; já nos assintomáticos o peso era de 117 kg e o IMC de 47 kg/m². Houve significância estatística na comparação entre indivíduos com e sem sintomas nas variáveis de peso inicial (p=0,00000), peso atual (p=0,00029), IMC pré-operatório (p=0,00219) e IMC pós-operatório (p=0,00148).

**Conclusão:**

A presença de sintomas compatíveis com tuba auditiva patente foi de 18,75% dos pacientes submetidos à cirurgia bariátrica na amostra avaliada. Tanto o peso pré-operatório, quanto o IMC foram menores nos pacientes sintomáticos, quando comparados com o grupo assintomático.

## INTRODUCTION

The Eustachian tube was firstly described in detail by Bartholomeus Eustachius in the 16^th^ century. This osteocartilaginous channel that communicates the middle ear with the rhinopharynx^1^ ^,^ ^10^ has, when in operating normaly, three important functions: equalization of pressure and ventilation of the middle ear, mucociliary clearance of middle ear secretions and protection of the middle ear against sounds, pathogens and secretions from the rhinopharynx^1^ ^,^ ^3^ ^,^ ^5^ ^,^ ^10^ ^,^ ^11^ ^,^ ^13^ ^,^ ^14^ ^,^ ^16^. 

The disease known as patulous Eustachian tube (PET) is defined as when the Eustachian tube remains permanently open^1^ ^,^ ^3^ ^,^ ^4^ ^,^ ^6^ ^,^ ^7^ ^,^ ^9^ ^,^ ^11^ ^,^ ^13^ ^,^ ^16^. In 1864, Schwartze described for the first time the movement of the tympanic membrane in sync with breathing^1^ ^,^ ^3^ ^,^ ^9^ ^,^ ^11^ ^,^ ^16^. Three years later, Jago first reported this syndrome, which he himself suffered from^1^ ^,^ ^3^. 

 PET is a rare entity in the general population, with an estimated prevalence between 0.3 and 6.6%; approximately 10-20% of patients with persistent complains^1^ ^,^ ^3^ ^,^ ^9^ ^,^ ^17^. It is more common in women and generally affects adolescents and adults, rarely being described in children^1^ ^,^ ^9^. The possible factors involved in its pathogenesis are the loss of the soft tissue that surrounds the cartilaginous portion of the auditory tube (Ostmann’s fatty layer), abnormal contractile muscle activity - tensor and elevator muscles of the soft palate and salpingopharyngeal muscle - and the inability of the pterygoid venous plexus in helping to close the auditory tube^4^ ^,^ ^9^ ^,^ ^10^. These factors may exist in the post-bariatric patient, and current data indicates that the prevalence of PET after bariatric surgery is more significant than in the general population.

This condition is associated with numerous causes, such as those that promote the loss of the layer of soft tissue that surrounds the auditory tube (pregnancy, use of oral contraceptives and use of estrogen), conditions that cause atrophy or fibrosis of the nasopharynx and its muscles (radiotherapy, polio, multiple sclerosis, stroke, temporomandibular disorder, iatrogenic trauma, palate myoclonus and craniofacial abnormalities) or that cause atrophy to the peritubary tissues (rheumatic diseases, allergic diseases and gastroesophageal reflux) ^1,3,4,6,8,9, 11,12,16^. Significant and rapid weight loss (as after bariatric surgery) can lead to a decrease in the soft tissue that surrounds the auditory tube and can be a risk factor for the development of PET^1,2,4,6,10,11,14, 16^.

PET patients may be asymptomatic or report symptoms from mild to severe ^2^ ^,^ ^11^. Most common complaints are autophony (subjective feeling of hearing your own voice when speaking), aural fullness, tinnitus and hearing your own breathing^1^ ^-^ ^3^ ^,^ ^6^ ^,^ ^7^ ^,^ ^9^ ^-^ ^11^ ^,^ ^13^ ^-^ ^16^. As an improvement factor there is supine position, placing the head between the knees, infections of the upper airways or performing reverse Valsalva maneuver^1^ ^,^ ^3^ ^,^ ^11^ ^,^ ^14^ ^,^ ^15^. As a worsening factor there is practice of exercises, prolonged use of the voice and taking nasal or oral decongestants ^3^ ^,^ ^11^ ^,^ ^14^.

The diagnosis is based on clinics, presence of risk factors, symptoms, and findings on physical examination such as movement of the tympanic membrane in sync with respiratory movements; although such movements may not be present in all patients^1^ ^-^ ^3^ ^,^ ^9^ ^,^ ^11^ ^,^ ^14^ ^,^ ^16^. Complementary methods such as computed tomography, magnetic resonance and nasal endoscopy can assist in diagnosis ^1^ ^,^ ^2^ ^,^ ^9^ ^,^ ^11^ ^,^ ^13^ ^,^ ^16^ ^,^ ^17^. Recently, there has been an increase in the recognition of this entity among clinicians, in addition to the advent of new diagnostic tools, which has resulted in an increase in its diagnosis and its related conditions^17^.

The association of weight loss and PET has been described in some reports of patients with anorexia and after bariatric surgery^1^ ^,^ ^2^ ^,^ ^6^ ^,^ ^9^ ^,^ ^10^ ^,^ ^16^. This corroborates the hypothesis that a decrease in the pressure of the peritubal tissue and loss of fatty deposit in the region of the auditory tube is important in the pathogenesis of PET.

There is a consensus that bariatric surgery is an effective and permanent treatment for clinically severe obesity, and as a result, the number of procedures performed has grown exponentially in recent years. There is evidence that due to the rapid and significant weight loss, these patients may be more likely to develop PET than the general population^9^ ^,^ ^10^. In the literature, there are few reports of clinical cases correlating significant weight loss and development of PET. 

The aim of this study was to determine the prevalence of auditory symptoms compatible with PET among patients after undergoing bariatric surgery and their relationship with initial weight, total weight loss and body mass index (BMI) and to verify possible clinical differences between those submitted to bariatric surgery who presented PET complaints and asymptomatic ones.

## METHODS

The present study was approved by the hospital ethics committee under number 46807115.6.0000.0096.

**FIGURE 1:**
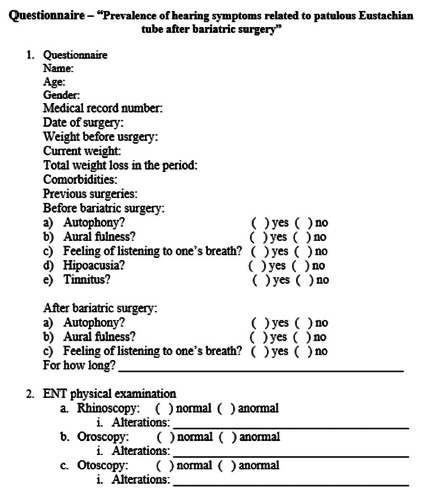
Questionnaire applied to study patients

 This was a cross-sectional study, which included patients who underwent Roux-en-Y gastric bypass (Fobi-Capella), with at least six months postoperative follow up, who answered a standardized questionnaire about the presence of symptoms compatible with PET before and after the operation (Figure 1). They were correlated to weight, initial BMI and at the time of assessment of those who had symptoms.

 As inclusion criteria, the following aspects were used: patients with at least six months postoperative follow up, with no distinction of gender, with an age range from 18-65 years. Those who underwent previous otological, adenoidectomy, and nasal operations, who presented with previous hearing deficits, previous neurological or rheumatological diseases were excluded. For the diagnosis of PET, positive answers to the questionnaire and the concomitant presence of autophony and aural fullness were considered.

### Statistical analysis

The chi-square test was used to compare weight and BMI for patients with and without symptoms with a 5% significance level (p=0,05).

## RESULTS

**FIGURE 2:**
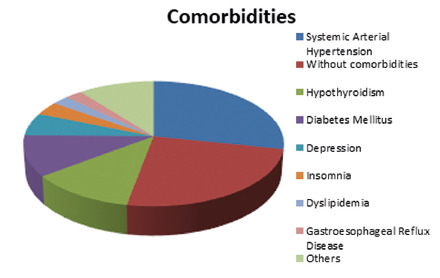
Comorbidities after bariatric surgery

During the period of 2015 to 2018, 80 patients were evaluated, mostly women (96.25%). Age varied between 23-72 years (mean 49.35), with postoperative follow up ranging from nine to 236 months (mean 60 months). About 66.25% of patients had at least one postoperative comorbidity, among them the most prevalent was systemic arterial hypertension (37.5%), followed by hypothyroidism (17.5%) and diabetes mellitus (13,75%, Figure 2).

 Regarding complaints presented in the postoperative period, there was a similar percentage of autophony, aural fullness and feeling of listening to one’s breathing (Table 1). The concomitant complaint of autophony and aural fullness was present in 15 patients (18.75%) compatible with clinical diagnosis of PET. Of those with symptoms, 53.33% presented otological alterations such as tympanic membrane retraction and areas of tympanosclerosis.

**TABLE 1:** Percentage of patients per symptom in the postoperative period

SYMPTOMS	YES (%)	NO (%)
Authopony	23 (29)	57 (71)
Aural fulness	33 (38)	47(62)
Feeling of listening to one's breath	23 (29)	57 (71)

 Among patients with symptoms, the initial weight was on average 112 kg and the BMI was 45 kg/m², whereas those who were asymptomatic had an average initial weight of 117 kg and a BMI 47 kg/m². There was a significant difference between the initial weight, current weight, pre- and postoperative BMI between patients with and without symptoms (Table 2).

**TABLE 2:** Comparison of patients with and without symptoms in relation to pre and postoperative BMI (kg/m²) and pre and postoperative weight (kg)

	With symptoms	Without symptoms	χ^2^ value
BMI preoperative	47	45	0,00219
Currente BMI	31	31	0,00148
Weight preoperative	117	112	0,00000
Current weight	79	77	0,00029

## DISCUSSION

The relationship between rapid and intense weight loss and the development of hearing symptoms resulting from PET - unable to maintain good functioning due to loss of its fatty tissue lining - is well described in the medical literature in patients with nervous anorexia^6^. Although weight loss is considered an important risk factor for the development of PET^1^ ^-^ ^3^ ^,^ ^9^ ^,^ ^11^ ^,^ ^14^ ^,^ ^16^ there is still no consistent relationship between the occurrence of this condition and performance of bariatric surgery.

The first case of PET in English literature was published in 2009^2^, occurring after bariatric surgery in a 44-year-old woman with a complaint of autophony and bilateral aural fullness. The authors concluded that PET may be one of the complications of surgery for obesity. Muñoz, Aedo and Der^9^ carried out a similar study, analyzing 141 patients who underwent bariatric surgery and observed PET in 21.28% of the patients, a percentage close to what was found in this series, although it was suggested that some of the symptoms were associated to other ear diseases, such as Menière, dehiscence of the upper semicircular canal or otosclerosis. 

As far as our research has reached, this is only the third work performed in Latin America that addresses PET after bariatric surgery. In this study, all patients with symptoms were women^9^ ^,^ ^10^; however, the majority of patients in the sample were women. It was also not possible to compare the speed of weight loss over time with the presence or absence of symptoms, due to the fact that this study is not longitudinal and there is great time variability between the date of the operation and the application of this questionnaire to the interviewees.

The existence of a significant difference in relation to the initial and current weight, and pre and postoperative BMI of patients with and without symptoms, had not yet been described in the literature. We found evidence that patients with symptoms compatible with PET have lower initial weight and BMI than those who do not fit this condition. Although we did not objectively measure Ostmann’s fat, we assume that patients with a higher initial weight would have more symptoms because they probably lost more of this tissue, so important in the support and competence of the auditory tube^2^ ^,^ ^9^ ^,^ ^10^, although this fact was not proven in our sample. 

In this study, we did not use otomicroscopic exams to check the movement of the tympanic membrane with breathing or other complementary exams to corroborate the diagnosis of PET. Thus, it is interesting that future studies use these resources in the search for a more reliable diagnosis of symptomatic patients. There is need for further studies to determine whether there is any compensatory mechanism in the face of the loss of the Ostmann fat layer that could explain the non-development of symptoms in part of patients undergoing bariatric surgery. Therefore, otorhinolaryngological support is important in the post-surgical management of these patients.

## CONCLUSION

The presence of symptoms resulting from patulous Eustachian tube (PET) was 18.75% among patients undergoing bariatric surgery. Preoperative weight and BMI were lower in symptomatic patients when compared to the asymptomatic group. 
